# Popliteal Neurovascular Bundle Medialization and Axial Flap Rotation to Avoid Bone Impingement in Tibial Turn-up Plasty: A Case Report

**DOI:** 10.1055/s-0044-1779308

**Published:** 2024-05-23

**Authors:** Manuel Ricardo Medellin Rincon, Oscar Fernando Gomez-Ricaurte, Hermann Alfredo Riveros-Riveros, Andrea Sambri

**Affiliations:** 1 Departmento de Cirurgia Ortopédica, Grupo Bogotano de Ortopedia y Sarcomas, Bogotá, Colômbia; 2 Departmento de Cirurgia Ortopédica, IRCCS Azienda Ospedaliera Universitaria di Bologna, Bolonha, Itália

**Keywords:** amputation stumps, bone transplantation, osteomyelitis, surgical flaps

## Abstract

A 33-year-old male patient developed distal femur chronic osteomyelitis with massive bone loss after an open grade-3b fracture. Following several failed treatments to eradicate infection, a tibial turn-up procedure was performed to provide a stable and functional stump. To avoid neurovascular problems, the popliteal vessels and sciatic nerve were moved medially, and the flap was rotated externally to decrease the collapse. The progression after surgery was satisfactory, no vascular or neurological claudication was observed, and the patient has been able to wear an external prosthesis after flap healing. Tibial turn-up plasty is a rarely described reconstructive technique capable of providing longer stumps. The releasing and medialization of popliteal vessels, with axial rotation of the flap, may prevent the development of neurovascular impingement.

## Introduction

Tibial turn-up is a uncommon type of amputation-rotationplasty that has several potential indications. 1 The objective of this technique is to develop a longer and functional stump, using the patient's leg or foot to reconstruct femoral or tibial defects. 2 Since the first report by Sauerbruch, 3 less than 30 cases have been published. It differs from a Van Nes rotationplasty 4 regarding the axis of rotation, as the leg is turned 180° over the thigh in the coronal plane instead of the axial plane.

Due to the very rare indication for this surgery, very little information has been written regarding the management of the neurovascular bundle. The purpose of the present paper is to describe an approach to the tibial turn-up technique to avoid impingement of the popliteal vessels and sciatic nerve.

## Case Report

Ethical clearance for the study was obtained from the institutional Ethics in Research Committee on September 12, 2022, and written informed consent was obtained from the patient.

A 33-year-old male patient sustained a Grade-IIIB open fracture of the distal femur and patella after a motorcycle accident in a rural area. He was initially treated in a low-complexity rural center through external fixation and several debridements due to gross contamination. As the process could not be managed locally, the patient was referred to our institution with a diagnosis of soft-tissue sepsis and osteomyelitis.

A distal femoral resection was required due to bone necrosis and persistence of the sepsis after wide-spectrum antibiotics and debridements. A temporary cement spacer was placed, and control of the septic process was achieved.

The wounds healed and the hardware remained stable during a six-week antibiotic course. Patellar osteosynthesis was performed to preserve the extensor mechanism. The patient remained with the fixator for six months due to loss to follow-up. At that point, the implants were removed and converted into a new spacer over a long intramedullary nail for arthrodesis.

**Fig. 1:**
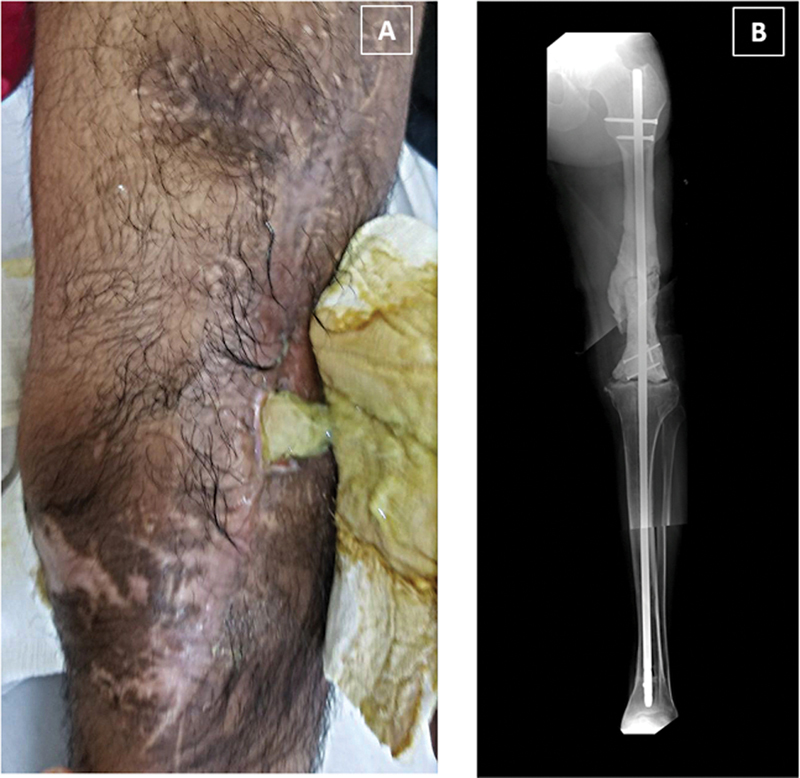
Clinical and radiological aspect one year after the knee arthrodesis procedure. ( **A** ) In the soft tissue, a fistula with purulent drainage can be observed. ( **B** ) Reconstructed anteroposterior panoramic X-ray view. The extension of the nail can be observed, including the changes in the femur, such as the sclerotic areas and the new bone formation around the cement spacer. In the distal tibia, radiolucent areas with relationship with the tip of the nail were identified.

The patient decided to continue with the spacer, but after one year the infection reactivated ( Fig. 1A ). Considering the difficulties to reconstruct the femur, soft-tissue compromise, and extension of the infection ( Fig. 1B ), the patient selected an ablative surgery. A tibial turn-up plasty was proposed to preserve length, following a debridement with samples, and a new antibiotic scheme.

**Fig. 2:**
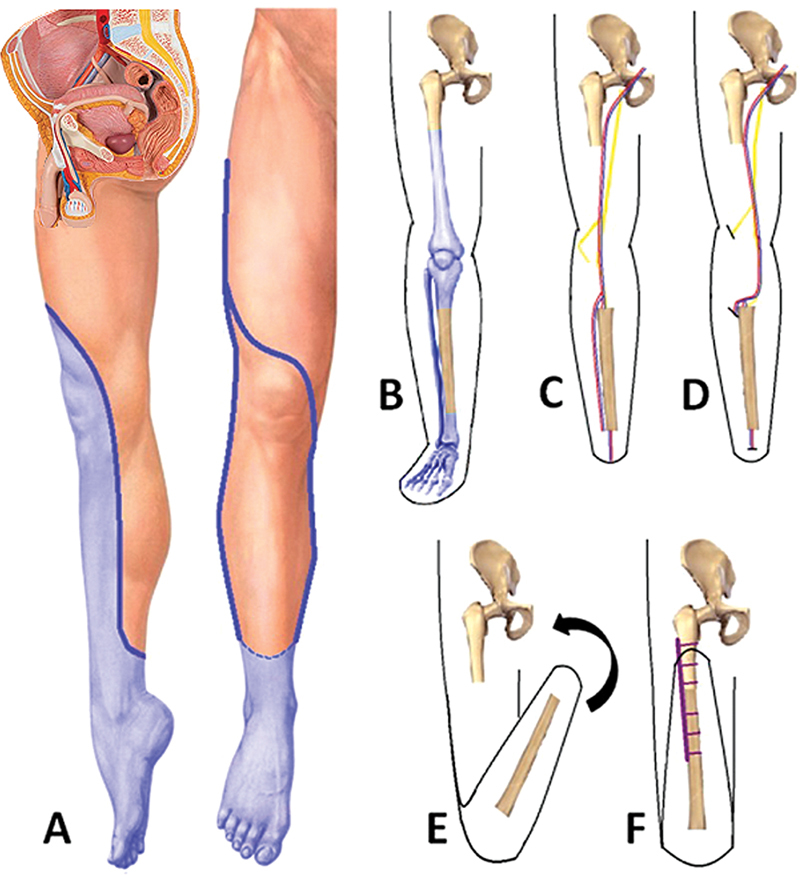
Summary of the steps involved in the surgical technique and intraoperative images. ( **A** ) Flap design on anteroposterior and medial views, indicating the areas to be resected. ( **B** ) In light blue color, the bone segments to be removed were identified. ( **C** ) Preliminary aspect of the flap after bone excision with the normal distribution of the neurovascular structures. ( **D** ) Medial displacement of the popliteal vessels and ligation of the anterior and posterior tibial vessels. The common peroneal nerve was also moved after fibula resection. ( **E** ) Tibial turn-up and length adjustment of the intercalary segment. ( **F** ) Internal fixation of the tibial and femoral components with a dynamic compression plate.

In the final surgery, the hardware was removed, and the tibial/femoral canals were cleaned using an irrigation-aspiration system (Reamer Irrigator Aspirator [RIA], Johnson & Johnson MedTech, New Brunswick, NJ, United States). The flap was designed, resecting the anterolateral thigh skin, patella, ligaments, proximal and distal thirds of the tibia, the fibula, and the anterior part of the skin of the leg skin and foot ( Fig. 2A ). The anterior tibial periosteum was preserved by removal of the tibialis anterior muscle ( Fig. 2B ).

**Fig. 3:**
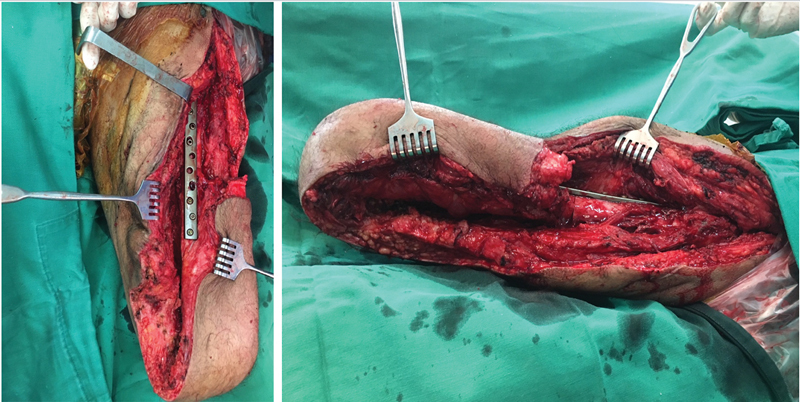
Intraoperative anteroposterior and lateral images displaying the osteomusculocutaneous flap once it was turned up and fixed to the femur.

The tibialis vessels were tied distally; the popliteal vessels and sciatic nerve were identified and medially moved in the flexion area of the flap ( Fig. 2C and 2D ). The remaining tibia was adjusted and fixed to the femur with a 4.5-mm titanium dynamic compression plate (DCP) (Königsee Implantate GmbH, Allendorf, Hessen, Germany), after rotating the flap 45° in the axial plane ( Figs. 2E and 3 ). The soft tissue and skin were closed in a normal fashion.

**Fig. 4:**
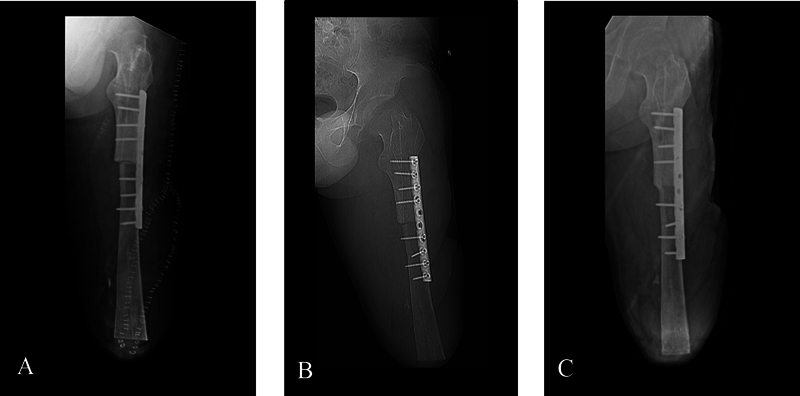
The sequence presents the progression of the consolidation between the femur and tibia. ( **A** ) The X-rays were taken in the immediate postoperative period. Skin clips can be observed in the wound area delimitating the flap. ( **B** ) Image acquired two months after surgery. Some callus formation was observed, but no solid findings of consolidation could be identified. ( **C** ) X-rays obtained after six months, showing complete consolidation at the junction.

**Fig. 5:**
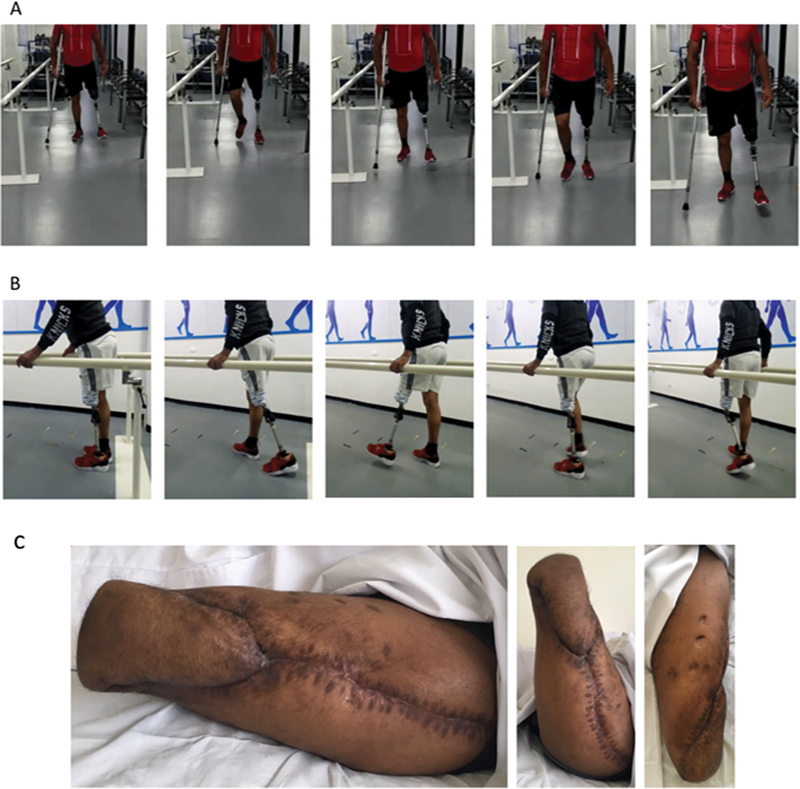
Final clinical aspect during gait rehabilitation with the use of external prosthesis. The patient is walking with partial weight bearing using one crutch. ( **A** ) Full weight bearing was performed in line with the lateral supports. ( **B** ) Photos of the stump once the wounds healed. ( **C** ) Lateral view of the two images on the left-hand side. The patient is performing active hip flexion and extension effortlessly.

An uneventful recovery was observed, with bone consolidation at six months, ( Fig. 4 ). The patient was followed up for more than 40 months. He is fully recovered and is able to perform his activities with an external prosthesis ( Fig. 5A and 5B ).

## Discussion

Tibial turn-up is a highly uncommon type of surgery performed when no other reconstructive alternatives are available. 5 Although it is an ablative intervention, its main objective is to develop longer amputation stumps, when total or almost total resection of the femur or tibia must be performed. 6

Due to the anatomical repairs in this reconstruction, the popliteal vessels and sciatic nerve will lie behind the tibial segment after the flap inversion. Therefore, there is a risk of impingement with vascular or neurological claudication following the prosthetic fitting.

Since the first report by Ferdinand Sauerbruch in 1922, 3 a total of 18 papers have been published in indexed databases. (PubMed, Hinari, ScienceDirect). The description of the proper management of the popliteal neurovascular bundle is available only in two 7 8 of those reports.

In the first one, Peterson et al. 7 mentioned that popliteal vessels may need to be freed for 8 cm to 10 cm at the point of rotation, to avoid tethering with the tibia. No further description was made regarding the neurovascular management.

In the second one, McDonald et al., 8 described a pedicled tibial bone flap without soft tissue. Although the usefulness of medializing the vascular component is mentioned in this paper, this technique depends on thigh flaps and sacrifices the sciatic nerve. Therefore, their observations cannot be evaluated in the conventional Sauerbruch reconstruction.

In the case herein reported, releasing the popliteal artery and sciatic nerve at the area of flexion enabled its medial displacement, avoiding impingement with the bone. Additionally, rotating the flap 45° in the axial plane increased the length of the vessel and nerve, decreasing the possible loop collapse.

In conclusion, tibial turn-up is a very useful but not frequently used last resource technique to reconstruct long femoral stumps. Although several modifications can be made, 9 the medialization and relative elongation of the neurovascular structures by rotating the flap seem to be a useful strategy to prevent bone impingement with early or late complications in the stump.
