# Supplementary material for: Popliteal Neurovascular Bundle Medialization and Axial Flap Rotation to Avoid Bone Impingement in Tibial Turn-up Plasty: A Case Report
Source: Rev Bras Ortop (Sao Paulo). 2024 May 23;59(Suppl 2):e163–7. doi: 10.1055/s-0044-1779308 (PMC11679619; doi:10.1055/s-0044-1779308)
Supplement: Related texts

# Research article: Medialização do feixe neurovascular poplíteo e rotação axial do retalho para evitar o impacto ósseo na plastia de inversão tibial: Relato de caso

**Keywords:** cotos de amputação, osteomielite, retalhos cirúrgicos, transplante ósseo

## Abstract

Um paciente do sexo masculino, de 33 anos, apresentou osteomielite crônica do fêmur distal com perda óssea maciça após fratura exposta de grau 3b. Após vários tratamentos ineficazes para erradicar a infecção, foi realizado um procedimento de aumento da tíbia para proporcionar um coto estável e funcional. Para evitar problemas neurovasculares, os vasos poplíteos e o nervo ciático foram movidos em sentido medial e o retalho foi rotacionado externamente para diminuição do colapso. A evolução após a cirurgia foi satisfatória; não houve claudicação vascular ou neurológica, e o paciente pôde usar prótese externa após a cicatrização do retalho. A plastia tibial é uma técnica reconstrutiva raramente descrita, que pode aumentar o comprimento dos cotos. A liberação e medialização dos vasos poplíteos, com rotação axial do retalho, pode prevenir o desenvolvimento de impacto neurovascular.

## Introdução

O *turn-up* tibial é um tipo incomum de amputação-rotoplastia com diversas possíveis indicações. 1 O objetivo desta técnica é o desenvolvimento de um coto mais longo e funcional, utilizando a perna ou o pé do paciente para a reconstrução de defeitos femorais ou tibiais. 2 Desde o primeiro relato de Sauerbruch, 3 menos de 30 casos foram publicados. A diferença com relação à rotoplastia de Van Nes 4 é o eixo de rotação, pois a perna é girada 180° sobre a coxa no plano coronal em vez de no plano axial.

Devido à raridade de indicação desta cirurgia, há poucas informações sobre o manejo do feixe neurovascular. O objetivo deste artigo é apresentar uma abordagem à técnica de *turn-up* tibial para evitar o impacto dos vasos poplíteos e do nervo ciático.

## Relato de Caso

Este relato de caso foi autorizado pelo Comitê de Ética institucional em 12 de setembro de 2022, e o paciente assinou o termo de consentimento livre e esclarecido.

Um paciente do sexo masculino, de 33 anos, sofreu uma fratura exposta de grau IIIB do fêmur distal e da patela após um acidente de moto em área rural. Foi atendido inicialmente em centro rural de baixa complexidade, onde foi submetido à fixação externa e a diversos desbridamentos por contaminação macroscópica. Como o caso não pôde ser tratado no local, o paciente foi encaminhado à nossa instituição com diagnóstico de sepse de partes moles e osteomielite.

Houve necessidade de ressecção femoral distal devido à necrose óssea e à persistência da sepse após a administração de antibióticos de amplo espectro e desbridamentos. O paciente recebeu um espaçador provisório de cimento e o processo séptico foi controlado.

As feridas cicatrizaram e o implante permaneceu estável durante o tratamento antibiótico por seis semanas. A osteossíntese patelar foi realizada para preservar o mecanismo extensor. O paciente continuou com o fixador por seis meses devido à perda de acompanhamento. Então, os implantes foram removidos e convertidos em um novo espaçador sobre uma haste intramedular longa para artrodese

O paciente decidiu continuar com o espaçador, mas, depois de um ano, houve reativação da infecção ( Fig. 1A ). Considerando as dificuldades de reconstrução do fêmur, o comprometimento de partes moles e a extensão da infecção ( Fig. 1B ), o paciente optou por cirurgia ablativa. Foi proposta uma plastia tibial para a preservação do comprimento após o desbridamento com coleta de amostras e novo esquema antibiótico.

**Fig. 1:** Aspecto clínico e radiológico um ano após o procedimento de artrodese de joelho. ( **A** ) Os tecidos moles apresentam fístula com secreção purulenta. ( **B** ) Radiografia panorâmica anteroposterior reconstruída. A extensão da haste pode ser observada, incluindo as alterações no fêmur, como as áreas escleróticas e a nova formação óssea em volta do espaçador de cimento. Na tíbia distal, foram identificadas zonas radiolúcidas relacionadas à ponta da haste.

Na última cirurgia, os implantes foram removidos e os canais tibiais/femorais foram limpos com sistema de irrigação-aspiração (Reamer Irrigator Aspirator [RIA], Johnson & Johnson MedTech, New Brunswick, NJ, Estados Unidos). O retalho foi desenhado, com ressecção da pele anterolateral da coxa, patela, ligamentos, terços proximal e distal da tíbia, fíbula, e parte anterior da pele da perna e do pé ( Fig. 2A ). O periósteo tibial anterior foi preservado removendo-se o músculo tibial anterior ( Fig. 2B ).

**Fig. 2:** Resumo das etapas cirúrgicas e imagens intraoperatórias. ( **A** ) Desenho do retalho nas incidências anteroposterior e medial, indicando as zonas a serem submetidas à ressecção. ( **B** ) A cor azul-claro identifica os segmentos ósseos a serem removidos. ( **C** ) Aspecto preliminar do retalho após excisão óssea com distribuição normal das estruturas neurovasculares. ( **D** ) Deslocamento medial dos vasos poplíteos e ligadura dos vasos tibiais anteriores e posteriores. O nervo fibular comum também foi movido após a ressecção da fíbula. ( **E** ) *Turn-up* tibial e ajuste do comprimento do segmento intercalar. ( **F** ) Fixação interna dos componentes tibial e femoral com placa de compressão dinâmica.

Os vasos tibiais foram ligados distalmente; os vasos poplíteos e o nervo ciático foram identificados e movidos medialmente na área de flexão do retalho ( Figs. 2C e 2D ). A tíbia remanescente foi ajustada e fixada ao fêmur com placa de compressão dinâmica (PCDD) de titânio de 4,5 mm (Königsee Implantate GmbH, Allendorf, Hessen, Alemanha) após a rotação do retalho em 45° no plano axial ( Figs. 2E e 3 ). O fechamento dos tecidos moles e da pele foi realizado da maneira habitual.

**Fig. 3:** Imagens intraoperatórias em incidência anteroposterior e de perfil mostrando o retalho osteomusculocutâneo virado para cima e fixado ao fêmur.

Não houve intercorrências à recuperação, e o paciente apresentou consolidação óssea aos seis meses ( Fig. 4 ). O acompanhamento foi mantido por mais de 40 meses. O paciente está totalmente recuperado, e é capaz de realizar suas atividades com prótese externa ( Figs. 5A e 5B ).

**Fig. 4:** A sequência apresenta a progressão da consolidação entre o fêmur e a tíbia. ( **A** ) As radiografias foram realizadas no período pós-operatório imediato. Há clipes cutâneos na área da ferida que delimita o retalho. ( **B** ) Imagem adquirida dois meses após a cirurgia. Há alguma formação de calo, mas nenhum achado sólido de consolidação pôde ser identificado. ( **C** ) Radiografias obtidas após seis meses, mostrando a consolidação completa na junção.

**Fig. 5:** Aspecto clínico final durante a reabilitação da marcha com uso de prótese externa. O paciente caminha com carga parcial, utilizando uma muleta. ( **A** ) A sustentação total do peso foi realizada alinhada aos apoios laterais. ( **B** ) Fotos do coto após a cicatrização das feridas. ( **C** ) Vista lateral das duas imagens do lado esquerdo. O paciente realiza flexão e extensão ativa do quadril sem esforço.

## Discussão

O *turn-up* tibial é um tipo de cirurgia muito incomum, realizado na ausência de alternativas reconstrutivas. 5 Embora seja uma intervenção ablativa, seu principal objetivo é o desenvolvimento de cotos de amputação mais longos nos casos de ressecção total ou quase total do fêmur ou da tíbia. 6

Os reparos anatômicos nesta reconstrução fazem com que os vasos poplíteos e o nervo ciático fiquem atrás do segmento tibial após a inversão do retalho. Portanto, há risco de impacto com claudicação vascular ou neurológica após a adaptação protética.

Desde o primeiro relato de Ferdinand Sauerbruch em 1922, 3 um total de 18 artigos foram publicados em bases de dados indexadas (PubMed, Hinari, ScienceDirect). O manejo adequado do feixe neurovascular poplíteo foi descrito em apenas em dois 7 8 desses estudos.

No primeiro, Peterson et al. 7 mencionaram que os vasos poplíteos podem precisar de liberação de 8 cm a 10 cm no ponto de rotação para evitar a amarração da tíbia. Nenhuma outra descrição foi feita sobre o manejo neurovascular.

No segundo, McDonald et al. 8 descreveram um retalho ósseo tibial pediculado sem partes moles. Embora este artigo mencione a utilidade da medialização do componente vascular, sua técnica depende de retalhos da coxa e sacrifica o nervo ciático. Portanto, suas observações não podem ser avaliadas na reconstrução convencional de Sauerbruch.

No caso aqui relatado, a liberação da artéria poplítea e do nervo ciático na área de flexão permitiu seu deslocamento medial, o que evitou o impacto com o osso. Além disso, a rotação do retalho em 45° no plano axial aumentou o comprimento do vaso e do nervo, e diminuiu o possível colapso da alça.

Em conclusão, o *turn-up* tibial é uma técnica de último recurso muito útil, mas pouco utilizada, para a reconstrução de cotos femorais longos. Embora diversas modificações possam ser feitas, 9 a medialização e o alongamento relativo das estruturas neurovasculares por meio da rotação do retalho parece ser uma boa estratégia para a prevenção do impacto ósseo e das complicações precoces ou tardias no coto.
